# Durable targeting of B-lymphocytes in living mice

**DOI:** 10.1038/s41598-018-29452-0

**Published:** 2018-07-24

**Authors:** M. Cascalho, D. Huynh, A. R. Lefferts, L. Stein, T. Lanigan, J. Decker, L. D. Shea, J. L. Platt

**Affiliations:** 1 Department of Microbiology & Immunology, University of Michigan, 1150 W, Medical Center Dr., Ann Arbor, MI 48109-5656 USA; 2 Department of Surgery, University of Michigan, 1150 W, Medical Center Dr., Ann Arbor, MI 48109-5656 USA; 3 Department of Medicine, University of Michigan, 1150 W, Medical Center Dr., Ann Arbor, MI 48109-5656 USA; 4 Department of Bioengineering, University of Michigan, 1107 carl A. Gerstacker Building, 2200 Bonisteel Blv., Ann Arbor, MI 48109-2099 USA

## Abstract

Transfer to and enduring expression of genes in B cells has proved a vexing challenge. We report here a novel method for the specific and durable targeting of B lymphocytes in living mice. The method involves generation of lentiviruses pseudotyped with an anti-CD19 antibody. CD19 targeting viruses injected in the spleen of living mice efficiently transduced B cells and plasma cells detected by flow cytometry analysis of GFP expression. Expression of the reporter gene could be detected in the intact animal by external imaging for more than a year and was enhanced by booster immunization. Our method thus enables the specific delivery, expression and localization by external imaging of exogenous genes to the B cells and plasma cells of living individuals.

## Introduction

Several difficult hurdles have precluded the specific transduction of primary B cells in intact individuals and achievement of long-lasting expression of the transduced genes. Although B cells can be transduced with retroviral or lentiviral vectors in cell cultures^1–3^, after transfer to isologous individuals, the transduced cells are rapidly cleared or persist at a very low frequencies in immune competent individuals^4^. One hurdle to primary B cell transduction and long lasting gene expression may be incompatibility between commonly used viral pseudotypes, particularly the vesicular stomatitis virus G glycoprotein (VSVG), and the B cell surface. Levy *et al*.^5^ developed baboon envelope pseudotyped vectors that transduced primary human B cells in culture more efficiently than VSVG expressing vectors but longevity of the transduced B cells was only studied in immunodeficient mice and only for brief periods (<30 days). Frecha *et al*.^6^ transduced quiescent B cells by expressing measles virus H and F glycoproteins on lentiviruses but the longevity of the transduced B cells was not examined in living hosts.

The hurdles to transduction of primary B cells and persistent expression of transduced genes could, in principle, be overcome by targeting lentiviruses to B cell surface molecules (such as CD20 or CD19) that mediate endocytosis upon antibody binding or during B cell activation^7^. Yang *et al*.^8^ targeted human B cells with lentiviral vectors expressing anti-hCD20 antibodies using an optimized ph-dependent fusogen derived from the Sindbis virus envelope glycoprotein, which might facilitate membrane fusion and Kneissl *et al*.^2^ targeted resting human B cells in culture using anti-hCD20 or anti-hCD19 pseudotyped lentiviruses. However, neither group reported direct delivery to B cells in living individuals and the duration of expression in immunocompetent hosts was never tested, for obvious reasons.

We reasoned that transduction of B cells in the living animal rather than *in vitro* might allow long-lived expression of transduced B cells in a physiologic manner, owing to either memory or homeostasis. Accordingly, we constructed a lentivirus displaying anti-murine CD19 on the outer membrane and tested whether the virus could transduce B cells in living mice. In principle, the approach would target naïve or memory B cells, which express CD19 but not plasma cells, which do not^9^.

## Results

### Anti-CD19 pseudotyped lentiviruses

**Figure 1:**
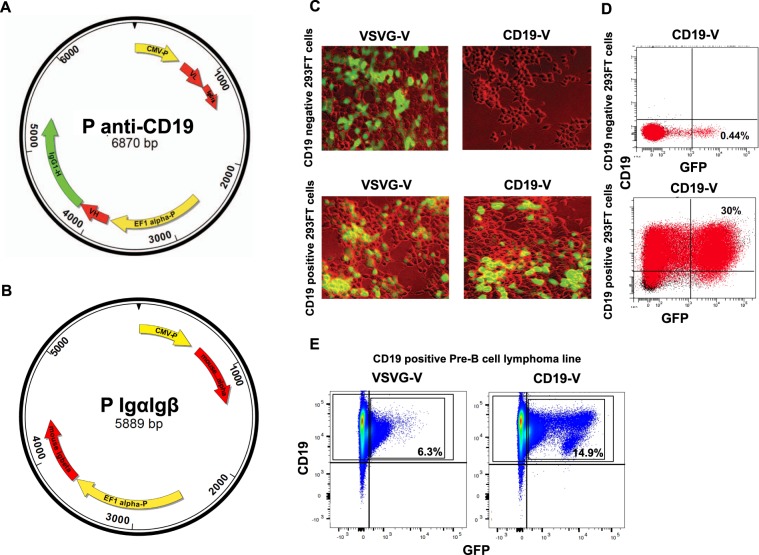
Lentiviral vectors pseudotyped with anti-CD19 antibodies efficiently target CD19-positive cells. (**A**) pαmCD19 plasmid map. The light chain and heavy chain variable regions of monoclonal rat anti-mouse CD19 were obtained by PCR from the clone 1D3 (ATCC). To generate pαmCD19, the full-length chimeric light chain and full-length heavy chain regions were cloned into pBudCE4.1 (Invitrogen) under control of the CMV promoter and the EF1α promoters, respectively. (**B**) pmIgαβ plasmid map. Genes encoding mouse Igα and Igβ, proteins required for expression of antibodies on the surface of cells, were amplified by RT-PCR and inserted into pBudCE4.1, designated pmIgαβ. (**C**–**E**) Lentiviral transduction of CD19−, CD19+ 293-FT cells or of 18.81 CD19+ Pre-B cells with anti-CD19 (CD19-V) or VSVG (VSVG-V) pseudotyped lentiviruses. Transduced cells can be identified by expression of GFP (green, C, or on the x-axis D and E). The figure shows that anti-CD19 pseudotyped viruses specifically transduce CD19+ cells, while control virus transduces (transduce intead of transduces) CD19− and CD19+ cells. (**D**) Shows flow cytometry analysis of CD19− and CD19+ 293FT cells transduced with anti-CD19 pseudotyped lentiviruses. Anti-CD19 pseudo-viruses transduce 293FT CD19+ cells >68 times more efficiently than 293FT CD19-negative cells. (**E**) Shows that CD19-V transduce Pre-B lymphoma cells expressing endogenous CD19, 2.4 more efficiently than VSVG-V at 72 hours post-transduction.

To enable B cell-specific targeting, we cloned cDNAs of the κ light chain constant region and the trans-membrane mouse IgG1 constant regions from the C57BL/6 mouse by amplification with AccuPrime *pfx* SuperMix (Invitrogen). The Ig light (L) chain and heavy (H) chain variable (V) regions from monoclonal rat anti-mouse CD19 antibody were cloned from 1D3 cells (ATCC) using GeneRacer kit (Invitrogen), fused in frame to the corresponding constant regions by overlapping PCR and cloned into the eukaryotic expression vector pBudCE4.1 (Invitrogen). In the pαmCD19, the full-length chimeric light chain was cloned 3′ of the CMV promoter and the chimeric heavy chain was cloned 3′ to the EF1α promoter (Fig. 1A). Sequencing confirmed that the inserts were complete and in frame (sequences are included as Supplementary data).

To pseudo type lentiviruses, proteins must be expressed on the surface of cells producing the virus. Cell surface display of antibodies requires expression of the products of the genes encoding Igα and Igβ (CD79, a and b), which assist in the assembly of the Ig on the surface of cells. Igα and Igβ form a disulfide-linked heterodimer that associates with the membrane Ig and constitute the signaling portion of the B cell receptor. We therefore cloned Igα and Igβ from C57BL/6 B cells using RT-PCR and inserted the complete cDNAs into pBudCE4.1; we designate the cDNA pmIgαβ (Fig. 1B). Sequences of pmIgαβ vectors were confirmed by sequencing and are provided as supplementary data.

To generate lentiviruses for delivery to B cells, 293FT cells (Invitrogen) were transfected with packaging vectors psPAX2, pSinmu^10^, pα-CD19, and pIgαβ and reporter proviral plasmids, pLentilox3.7 (GFP) or pLentilox-luciferase (UM Vector Core) using standard PEI precipitation methods. The viral supernatants were harvested 72 h after transfection, concentrated by centrifugation and stored at −80 °C until use. The efficiency of transduction was determined by transduction of 0.2 × 10^6^ 293/CD19+ cells by spin-infection with 0.5 ml of serially diluted viral supernatants. The transduction titer (TT) measured after 3 days culture at 37 °C was calculated by enumerating GFP-positive cells at dilution ranges exhibiting a linear dose response. The transduction units (TU) reflect the number of GFP-positive cells for each unit of virus volume, typically >10^8^ per ml.

To test the specificity of the anti-CD19 typed lentivirus, we transduced 2 × 10^6^ CD19+ and 2 × 10^6^ CD19− 293FT cells by spin infection with 10^7^ transducing units of concentrated anti-CD19 psudotyped virus or with VSVG pseudotyped control viruses and assayed expression by flow cytometry and immunofluorescence. Figure 1C, and D show that anti-CD19 pseudotyped viruses only transduce CD19 + cells while VSVG pseudotyped viruses transduce CD19− and CD19+ cells. We determined that anti-CD19 antibody pseudotyped lentivirus targeted cells expressing endogenous CD19 by transducing murine pre-B lymphoma cells (18.81^11^). As Fig. 1E shows, the anti-CD19 pseudotyped viruses transduce CD19+ B cells at least 2 times more efficiently than VSVG pseudo typed viruses.

### *In vivo* B cell transduction

**Figure 2:**
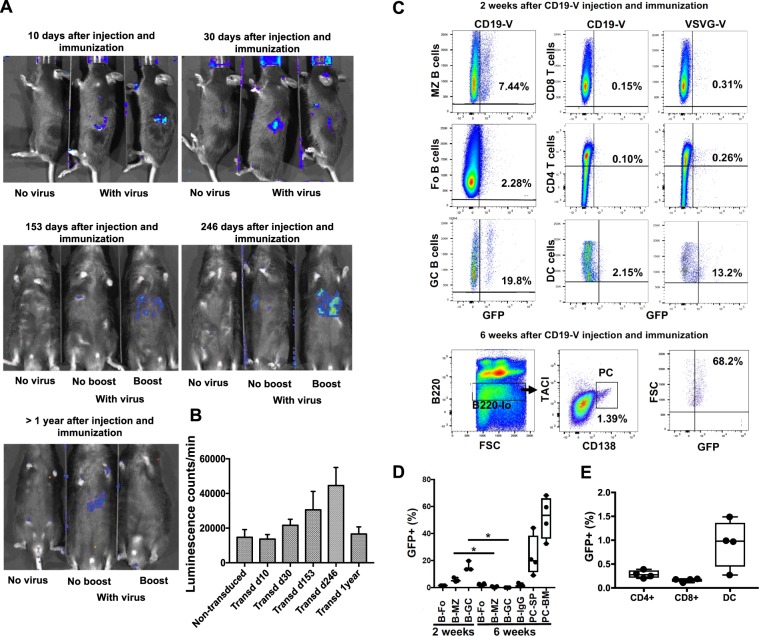
Enduring expression of vectors delivered by anti-CD19 pseudotyped lentivirus in living mice. (**A**) Luciferase activity was determined 5 minutes after injection of 2 mg of D-lucipherin in 200 μl PBS/mouse (Promega) (*i.p*) at various times after injection of anti-CD19 pseudotyped lentivirus into the spleen of mice. The mice were immunized with sheep red blood cells (SRBC) 3 days before injection of the lentivirus. Figure shows luciferase activity measured at 10 days, 30 days, 153 days, 246 days and at a year after transduction. Mice were boosted with SRC 10 days before analysis at time points later than 1 month, as indicated. The figure depicts images typical 3 or more independent experiments. (**B**) Luciferase activity was determined 5 minutes after injection of 2 mg of D-lucipherin in 200 μl PBS/mouse (Promega) (*i.p*) at various times after injection of anti-CD19 pseudotyped lentivirus into the spleen of mice. Figure shows luciferase activity measured at 10 days, 30 days, 153 days, 246 days and at a year after transduction. The figure shows graph depicting the average of two of more measurements of luminescence captured by a Xenogen IVIS 200 imaging system in live mice. (**C–E**) Flow cytometry analysis of splenocytes obtained from mice 2 or 6 weeks after intra-splenic injection of anti-CD19 (or VSVG) pseudotyped virus expressing a GFP reporter. The mice were immunized with SRBC 3 days before injection of the lentivirus and for the 6 weeks analysis boosted 10 days prior. Anti-CD19 pseudotyped lentivirus efficiently transduced B cells. At two weeks almost 20% of the GC B cells expressed GFP and at 6 weeks more than 50% of plasma cells in the bone marrow did. (**D**, **E**) Depict the frequencies of GFP+ B cell subsets at 2 and 6 weeks post transduction with CD19-V (**D**), and of GFP+ non-B cells at 6 weeks post-transduction with CD19-V (**E**).

To determine if anti-CD19 pseudotyped virus could target B cells in living mice, we devised one vector employing luciferase as the reporter and another GFP. Mice immunized 3 days before with sheep red blood cells, were anesthetized and then administered 10^8^ transduction units of CD19-pseudotyped lentiviruses by direct injection into the spleen. We injected lentiviruses in the spleen rather than i.v. to allow injection of volumes in excess of 100 μl. Figure 2A shows that after intra-splenic injection of the pseudotyped virus with a luciferase reporter construct, luciferase emission can be detected and localized by external imaging for more than one year. Figure 2B shows that from the time of injection, luciferase expression gradually increases up to 246 days and declines thereafter. At 153 days and 246 days following lentiviral injection luciferase expression was enhanced by booster immunization with 10^8^ sheep red blood cells but not after one year. Figures 2C, and D show that 2 weeks after intra-splenic injection of the anti-CD19 pseudotyped virus with the GFP reporter construct, GFP emission is detected mainly in germinal center B cells (CD19+, GL7+); whereas 6 weeks after injection GFP emission is detected mainly in plasma cells which do not express CD19 (B220 lo, CD138+, TACI+). In fact, at the 6 week time point, >50% of the bone marrow plasma cells (51.9%, on average) expressed GFP (GFP+ gates were set with non-transduced cells, Supplementary Fig. 2). This finding indicates that the anti-CD19 pseudotyped lentiviral vector is incorporated by B cells responding to antigen stimulation and the lentivirus is retained during proliferation and differentiation. Targeting was specific since few CD4+ or CD8+ T cells or dendritic cells expressed GFP (presence in a few dendritic cells could reflect phagocytosis of apoptotic B cells, Figs. 2C, and E). In contrast, a VSVG-pseudotyped lentivirus transduced all of these cells generating notable expression of GFP in non-B cells (Fig. 2C).

The targeting of B cells in living mice with the anti-CD19 pseudotyped lentiviruses had no apparent toxic effects. Treated mice remained healthy throughout the duration of the experiment and no tumors were observed upon euthanasia and necropsy.

## Discussion

Our findings show for the first time that B cells of living animals can be specifically targeted with a lentivirus construct and enduring expression can be achieved. Specific targeting is achieved by pseudotyping with anti-CD19 antibody and enduring expression during the course of B cell proliferation and differentiation is achieved using a lentiviral vector with fusion competent Sindbis envelope glycoprotein. Because the construct persists with B cell replication, the approach we report might be used to examine the frequency and fate of B cells of any given specificity or any antigen responsive sub-population. Since our construct can be easily modified to swap pseudotype specificities, the approach we describe might also be used to target any cell for which a specific surface marker has been identified.

## Methods

### Mice

C57BL/6 mice were purchased from the Jackson Laboratories. Male or female mice were 6 to 8 weeks old at the beginning of experiments and kept in a SPF facility throughout the length of observation period. All procedures were approved by the Institutional Animal Care and Use Committee at the University of Michigan.

### Vector construction

#### Vector pαmCD19 construction

A schematics of the vector pαmCD19 encoding anti-mouse CD19 antibody is shown in Fig. 1. The cDNA of the mouse κ light chain constant region and the membrane bound mouse IgG1 constant region were amplified from C57BL/6 mouse primary B cells with primer sets mouse IgκC-F/IgκC-R and mouse-IgG1-HC-F/IgG1-HC-R, respectively, using AccuPrime pfx SuperMix (Invitrogen). The light and heavy chain variable regions from rat anti-mouse CD19 antibody [clone 1D3 (ATCC)] were obtained using GeneRacer kit (Invitrogen) with primer sets Rat-Igκ-V-forward/Rat-Igκ-V-reverse and Rat-IgG2a-HV-F/Rat-IgG2a-HV-R, respectively. To generate rat/mouse chimeric light chain and heavy chain encoding regions, the amplicons were fused in frame to the corresponding constant regions by overlapping PCR using primer sets Rat-IgκV-F/ mouse IgκC-R and rat-IgG2a-HV-F/ mouse-IgG1-HC-R.

To generate pαmCD19, the full-length chimeric light chain and heavy chain fragments were cloned into pBudCE4.1 (Invitrogen) under the transcriptional control of the CMV and EF1α promoters, respectively.

**Rat-IgG2a-HV-F:** 5′-ACTGGGTACCGCCACCATGAGCACTATTTTCTCTATAG-3′

**Rat-IgG2a-HV-R:** 5′-aTGGGGGTGTCGTTTTGCTGAGGAGACTGTGACCA-3′

**mouse-IgG1-HC-F:** 5′-TCTCCTCAGCAAAACGACACCCCCATCTGT-3′

**mouse-IgG1-HC-R:** 5′-GTTGAGATCTCTAGGGCGCTTGCCCAATCA-3′

**Rat-Igκ-V-forward:** 5′-GTTGAAGCTTGCCACCATGGGTGTGCCCACTCAGCT-3′

**Rat-Igκ-V-reverse**: 5′-AGCATCAGCCGTACGTTTCAATTCCAGCTTGGT-3′

**mouse-IgκC-F:** 5′-GAAACGTACGGCTGATGCTGCACCAACT-3′

**mouse-IgκC-R:** 5′-GGGGACGCTCTAGATCAACACTCATTCCTGTTGA-3′.

#### Cloning of the vector pmIgαβ expressing mouse Igα and Igβ

Genes encoding mouse Igα and Igβ, which are required for plasma membrane expression of Ig and BCR signaling, were amplified from C57BL/6 B cells with primer sets Ig alpha–forward/Ig-alpha–reverse and Ig-beta-forward/Ig-beta-reverse, respectively, by using RT-PCR and inserted into pBudCE4.1. Fidelity of the cloned constructs was confirmed by sequencing and designated pmIgαβ.

**Ig-alpha-forward:** 5′-CATAAGCTTGCCACCATGCCAGGGGGTCTAGAAG-3′

**Ig-alpha-reverse:** 5′-ACAGGATCCTCATGGCTTTTCCAGCTGGGCATCT-3′

**Ig-beta-forward**: 5′-TAAGCGGCCGCCACCATGGCCACACTGGTGCTGT-3′

**Ig-beta-reverse:** 5′-GGACTCGAGTCATTCCTGGCCTGGATGCTCT-3′.

### Cloning of pcDNA-CD19-zeo

The CD19 cDNA was amplified from 18.81 pre-B cells with CD19-forward and CD19-reverse primers using AccuPrime pfx SuperMix (Invitrogen) and cloned downstream of the CMV promoter in pcDNA3.1-zeo to yield pcDNA-CD19-zeo.

**CD19 forward:** 5′-ACTGCTAGCGCCACCATGCCATCTCCTCTCCCTGTCT-3′

**CD19 reverse**: 5′-GGGTCTAGATCACGTGGTTCCCCAAGTC-3′.

### Cell Culture

HEK 293-FT cells from ATCC (PTA-5077) were cultured in DMEM with 10% fetal bovine serum, antibiotics and L-glutamine. HEK 293-FT cells expressing CD19 were cultured in the same medium with 100 µg/ml zeocin added. Pre-B cell line (18.81) was obtained from Dr. M. Wabl (University of California San Francisco, San Francisco, CA), and cultures in RPMI 1640 media (Gibco^TM^) with heat inactivated 10% fetal bovine serum, antibiotics (100U/ml penicillin and 100 microg/ml streptomycin, Gibco^TM^), L-glutamine and 2-beta-mercaptoethanol (5.5 microM, Gibco^TM^). The 18.81 B cells express CD19 on the surface, but lack surface expression of Ig receptor. !8.81 cell identity was checked by PCR of the characteristic VHDJH gene rearrangement.

### Lentivirus Production and Transduction

To produce the CD19-targeted lentiviruses, psPAX2 (228.75 µg), pSinmu (228.75 µg), pαmCD19 (152.5 µg), and pmIgαβ (152.5 µg) were co-transfected with 457.5 µg of pLentilox3.7 (GFP) or pLentilox-luciferase proviral plasmids by standard PEI precipitation. PEI precipitation was performed by incubating the plasmids with 4.8 mg PEI (molecular weight 2500, Polysciences, Inc) in 100 ml Optimum (Life technologies) for 20 min at 37 °C and then suspended in 900 ml DMEM with 10% FBS. The suspension was transferred to chambers of Corning CellSTACK each containing HEK 293-FT cells and the stacks were incubated at 30 °C for 72 hours. After incubation, the supernatants were collected, pooled, and filtered through 0.45 micron HV-Durapore Stericups (Millipore). The filtrates were, pelleted by centrifugation at 13,000 rpms in a Beckman Avanti J-E centrifuge at 4 °C for 4 hrs and then re-suspended at 100× the original concentration (~1 × 10^8^ TU/ml) in DMEM (Life technologies). The lentiviruses were stored in aliquots at −80 °C. The lentivirus-GFP-VSVG was provided as a stock virus by the University of Michigan Vector Core.

HEK 293-FT cells, CD19 expressing HEK 293-FT or 18.81 cells growing exponentially were transduced with anti-CD19 or VSVG pseudotyped lentivirus. Fifteen microliters of 100× virus (3 MOI) and 8 µg/ml Polybrene (Sigma) were added to 1.5 ml cell suspension and centrifuged at 2,500 rpm for 30 minutes at 37 °C. The cells were then incubated for 72 hr at 37° C, 5% CO_2_. Vector sinMu was a generous gift from Dr. David Baltimore^10^.

### Transduction of CD19+ Cells *in vivo*

One hundred microliters of concentrated lentiviruses (3xMOI) were injected directly into the spleen (visualized and mobilized through laparotomy) in mice that had been immunized *i.p*. with 2.5 × 10^8^ sheep red blood cells (Innovative Research, Novi, MI) 3 days before. The mice were given booster doses of SRBC *i.p*. 30 days later.

### Flow cytometry

Splenocytes were stained with combinations of rat monoclonal antibodies, according to established methods^12^. Anti-mCD19 (ID3), anti-mCD21(7G6), anti-mCD23(B3B4), anti-mIgD (26 c.2a), anti-mGL7 (GL7), anti CD45-R, B220 (RA3-6B2), anti-mTACI (ebio 8F10-3), anti-Dendritic Cell (33D1); anti-CD4 (RM4-5), anti-CD8 (53-6.7) were purchased from Invitrogen–Fisher Scientific, Pittsburgh, PA; or with anti-CD11b (M170); anti-CD138 (281.1); goat anti-mouse IgG (Poly 4053), purchased from Biolegend, San Diego, CA. Antibodies were conjugated to allophycocyanin (APC), phycoerythrin (PE), PerCP-Cy5.5, PE-Cy7 or biotin. Biotin conjugated antibodies were detected with Streptavidin conjugated to APC or PE, purchased from Invitrogen–Fisher Scientific Pittsburgh, PA. Marginal Zone B cells were CD19+, CD21 hi, CD23lo; Follicular B cells were CD19+, CD21+, CD23hi; Germinal Center B cells were CD19+, GL7+, IgD−; CD4+ T cells were CD19−. CD4+; CD8 T cells were CD19−, CD8+; Dendritic cells were CD19−, CD11b−, DC+; IgG+ B cells were CD19+, IgD−, IgG+; Plasma cells were B220lo, CD138+ and TACI+. Gates for GFP+ cells were set on equivalent populations obtained from non-transduced mice.

Six-color flow cytometric analysis of 10^6^ cells was performed using a FACSCanto II (BD Biosciences, San Jose, CA). GFP was read in the FITC channel. Analysis of plots was performed on live singlets by using FLOWJO, LLC 10.0.8 software.

### Imaging

Luciferase activity was imaged using a Xenogen IVIS 200 imaging system at the Center for Molecular Imaging at the University of Michigan Medical School in live mice injected with 100 microliters of 2 mg luciferin D (Promega) in PBS.

### Statistics

Results were analyzed by GraphPad Prism7 using two-tailed Mann-Whitney tests.

### Data availability

All data generated or analyzed during this study are included in this published article (and its Supplementary Information files).

### Ethical approval

The research reported involves mice. All experimental protocols were approved by the Institutional Animal Care and Use Committee at the University of Michigan and carried according with guidelines set forward by the Unit for Laboratory Animal Medicine at the University of Michigan.

## Electronic supplementary material

Supplemental Information
