# Supplementary material for: Durable targeting of B-lymphocytes in living mice
Source: Sci Rep. 2018 Jul 24;8:11143. doi: 10.1038/s41598-018-29452-0 (PMC6057982; doi:10.1038/s41598-018-29452-0)
Supplement: Supplementary file 1 — Supplemental Information [file 41598_2018_29452_MOESM1_ESM.pdf]

TITLE:

Durable targeting of B-lymphocytes in living mice

[1,2] M. Cascalho*, [2] D. Huynh, [2] A.R. Lefferts, [2] L. Stein, [3] T. Lanigan , [4] J. Decker, [4] L.D. Shea, and [1,2] J. L. Platt*

Supplemental materials

Supplemental Figure 1:

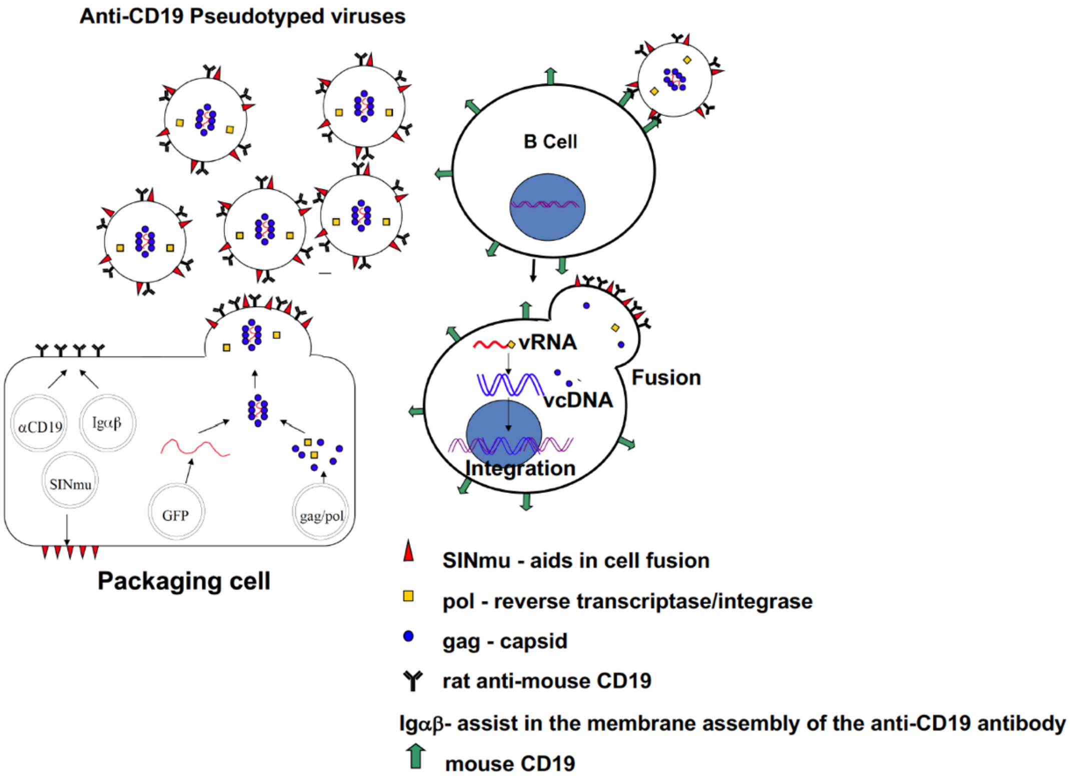

Supplemental Figure 1:

Schematics depicting CD19-V lentiviral synthesis followed by virus targeting and fusion with B cells. To generate lentiviruses for delivery to B cells, 293FT cells were transfected with packaging vectors psPAX2 encoding gag/pol, pSinmu, pα-CD19, pIgαβ and reporter proviral plasmids, pLentilox3.7 (GFP). 10^8 transduction units of CD19-pseudotyped lentiviruses were administered by direct injection into the spleen. Transduced B cells were identified by GFP expression detected by flow cytometry.

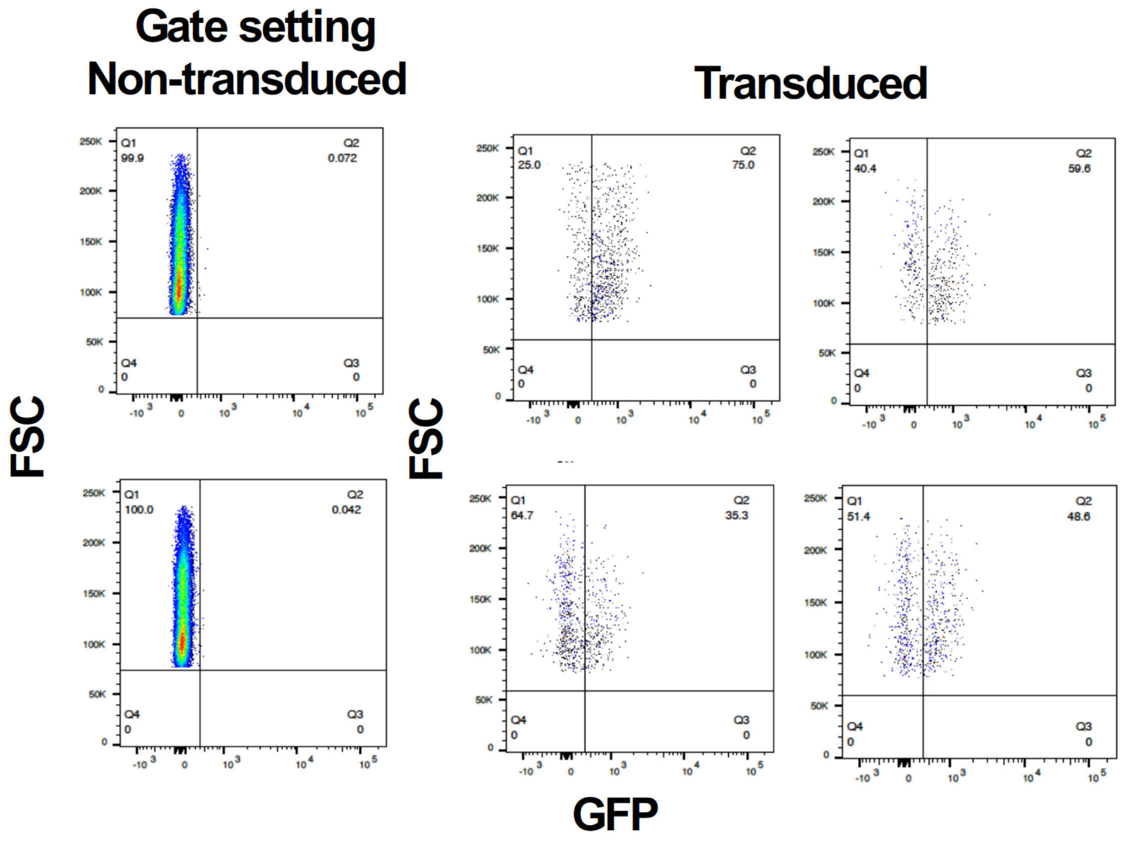

Supp. Figure 2

Supplemental Figure 2:

Flow cytometry plots of non-transduced or transduced bone marrow cells showing how the gate defining GFP+ cells was defined. Plots on the right depict B220lo, TACI+ and CD138+ plasma cells obtained from 4 different mice.

Supplemental Information

Sequence Information:

pαmCD19: Sequence that corresponds to map shown in figure 1A. In capital letters are depicted the VL, Cκ VH and Cγ1 exons.

```
5′ gcgcgcgttgacattgattattgactagttattaatagtaatcaattacggggtcattagttcatagcccatatatggag
ttccgcgttacataacttacggtaaatggcccgcctggctgaccgcccaacgacccccgcccattgacgtcaataatgac
gtatgttcccatagtaacgccaatagggactttccattgacgtcaatgggtggactatttacggtaaactgcccacttgg
cagtacatcaagtgtatcatatgccaagtacgccccctattgacgtcaatgacggtaaatggcccgcctggcattatgcc
cagtacatgaccttatgggactttcctacttggcagtacatctacgtattagtcatcgctattaccatggtgatgcggtt
ttggcagtacatcaatgggcgtggatagcggtttgactcacggggatttccaagtctccaccccattgacgtcaatggga
gtttgttttggcaccaaaatcaacgggactttccaaaatgtcgtaacaactccgccccattgacgcaaatgggcggtagg
cgtgtacggtgggaggtctatataagcagagctctctggctaactagagaacccactgcttactggcttatcgaaattaa
tacgactcactatagggagacccaagcttGCCACCATGGGTGTGCCCACTCAGCTCCTGGGGTTGTTGCTGCTGTGGATT
ACAGATGCCATATGTGACATCCAGATGACACAGTCTCCAGCTTCCCTGTCTACATCTCTGGGAGAAACTGTCACCATCCA
ATGTCAAGCAAGTGAGGACATTTACAGTGGTTTAGCGTGGTATCAGCAGAAGCCAGGGAAATCTCCTCAGCTCCTGATCT
ATGGTGCAAGTGACTTACAAGACGGCGTCCCATCACGATTCAGTGGCAGTGGATCTGGCACACAGTATTCTCTCAAGATC
ACCAGCATGCAAACTGAAGATGAAGGGGTTTATTTCTGTCAACAGGGTTTAACGTATCCTCGGACGTTCGGTGGCGGCAC
CAAGCTGGAATTGAAAcgtacgGCTGATGCTGCACCAACTGTATCCATCTTCCCACCATCCAGTGAGCAGTTAACATCTG
GAGGTGCCTCAGTCGTGTGCTTCTTGAACAACTTCTACCCCAAAGACATCAATGTCAAGTGGAAGATTGATGGCAGTGAA
CGACAAAATGGCGTCCTGAACAGTTGGACTGATCAGGACAGCAAAGACAGCACCTACAGCATGAGCAGCACCCTCACGTT
GACCAAGGACGAGTATGAACGACATAACAGCTATACCTGTGAGGCCACTCACAAGACATCAACTTCACCCATTGTCAAGA
GCTTCAACAGGAATGAGTGTTGAgtcgacatcgatcttaagcagtacttctagaggatccgaacaaaaactcatctcaga
agaggatctgaatatgcataccggtcatcatcaccatcaccattgagtttgatccccgggaattCAGACATGATAAGATA
CATTGATGAGTTTGGACAAACCACAACTAGAATGCAGTGAAAAAAATGCTTTATTTGTGAAATTTGTGATGCTATTGCTT
TATTTGTAACCATTATAAGCTGCAATAAACAAGTTggggtgggcgaagaactccagcatgagatccccgcgctggaggat
catccagccggcgtcccggaaaacgattccgaagcccaacctttcatagaaggcggcggtggaatcgaaatctcgtagca
cgtgtcagtcctgctcctcggccacgaagtgcacgcagttgccggccgggtcgcgcagggcgaactcccgcccccacggc
tgctcgccgatctcggtcatggccggcccggaggcgtcccggaagttcgtggacacgacctccgaccactcggcgtacag
ctcgtccaggccgcgcacccacacccaggccagggtgttgtccggcaccacctggtcctggaccgcgctgatgaacaggg
tcacgtcgtcccggaccacaccggcgaagtcgtcctccacgaagtcccgggagaacccgagccggtcggtccagaactcg
accgctccggcgacgtcgcgcgcggtgagcaccggaacggcactggtcaacttggccatggtttagttcctcaccttgtc
gtattatactatgccgatatactatgccgatgattaattgtcaacacgtgctgatcagatccgaaaatggatatacaagc
tcccgggagctttttgcaaaagcctaggcctccaaaaaagcctcctcactacttctggaatagctcagaggcagaggcgg
cctcggcctctgcataaataaaaaaaattagtcagccatggggcggagaatgggcggaactgggcggagttaggggcggg
atgggcggagttaggggcgggactatggttgctgactaattgagatgcatgctttgcatacttctgcctgctggggagcc
tggggactttccacacctggttgctgactaattgagatgcatgctttgcatacttctgcctgctggggagcctggggact
ttccacaccctcgtcgagctagcttcgtgaggctccggtgcccgtcagtgggcagagcgcacatcgcccacagtccccga
gaagttggggggaggggtcggcaattgaaccggtgcctagagaaggtggcgcggggtaaactgggaaagtgatgtcgtgt
actggctccgcctttttcccgagggtgggggagaaccgtatataagtgcagtagtcgccgtgaacgttctttttcgcaac
gggtttgccgccagaacacaggtaagtgccgtgtgtggttcccgcgggcctggcctctttacgggttatggcccttgcgt
gccttgaattacttccacctggctccagtacgtgattcttgatcccgagctggagccaggggcgggccttgcgctttagg
agccccttcgcctcgtgcttgagttgaggcctggcctgggcgctggggccgccgcgtgcgaatctggtggcaccttcgcg
cctgtctcgctgctttcgataagtctctagccatttaaaatttttgatgacctgctgcgacgctttttttctggcaagat
agtcttgtaaatgcgggccaggatctgcacactggtatttcggtttttgggcccgcggccggcgacggggcccgtgcgtc
ccagcgcacatgttcggcgaggcggggcctgcgagcgcggccaccgagaatcggacgggggtagtctcaagctggccggc
ctgctctggtgcctggcctcgcgccgccgtgtatcgccccgccctgggcggcaaggctggcccggtcggcaccagttgcg
tgagcggaaagatggccgcttcccggccctgctccagggggctcaaaatggaggacgcggcgctcgggagagcgggcggg
tgagtcacccacacaaaggaaaagggcctttccgtcctcagccgtcgcttcatgtgactccacggagtaccgggcgccgt
ccaggcacctcgattagttctggagcttttggagtacgtcgtctttaggttggggggaggggttttatgcgatggagttt
ccccacactgagtgggtggagactgaagttaggccagcttggcacttgatgtaattctcgttggaatttgccctttttga
gtttggatcttggttcattctcaagcctcagacagtggttcaaagtttttttcttccatttcaggtgtcgtgaacacgtg
gtcgcggccgcttcgaaggtaccGCCACCATGAAATGCAGCTGGATCATCCTCTTCTTGATGGCACTGGCTACAGGGGTC
AACTCAGAAGTCCAGCTGCAGCAGTCTGGGGCTGAGCTTGTGAGACCTGGGACCTCTGTGAAGTTATCTTGCAAAGTTTC
TGGCGATACCATTACATTTTACTACATGCACTTTGTGAAGCAAAGGCCTGGACAGGGTCTGGAATGGATAGGAAGGATTG
ATCCTGAGGATGAAAGTACTAAATATTCTGAGAAGTTCAAAAACAAGGCGACACTCACTGCAGATACATCTTCCAACACA
GCCTACCTGAAGCTCAGCAGCCTGACCTCTGAGGACACTGCAACCTATTTTTGTATCTACGGAGGATACTACTTTGATTA
CTGGGGCCAAGGGGTCATGGTCACAGTCTCCTCAAAAACGACACCCCCATCTGTCTATCCACTGGCCCCTGGATCTGCTG
CCCAAACTAACTCCATGGTGACCCTGGGATGCCTGGTCAAGGGCTATTTCCCTGAGCCAGTGACAGTGACCTGGAACTCT
GGATCCCTGTCCAGCGGTGTGCACACCTTCCCAGCTGTCCTGGAGTCTGACCTCTACACTCTGAGCAGCTCAGTGACTGT
CCCCTCCAGCCCTCGGCCCAGCGAGACCGTCACCTGCAACGTTGCCCACCCGGCCAGCAGCACCAAGGTGGACAAGAAAA
TTGTGCCCAGGGATTGTGGTTGTAAGCCTTGCATATGTACAGTCCCAGAAGTATCATCTGTCTTCATCTTCCCCCCAAAG
CCCAAGGATGTGCTCACCATTACTCTGACTCCTAAGGTCACGTGTGTTGTGGTAGACATCAGCAAGGATGATCCCGAGGT
```

```
CCAGTTCAGCTGGTTTGTAGATGATGTGGAGGTGCACACAGCTCAGACGCAACCCCGGGAGGAGCAGTTCAACAGCACTT
TCCGCTCAGTCAGTGAACTTCCCATCATGCACCAGGACTGGCTCAATGGCAAGGAGTTCAAATGCAGGGTCAACAGTGCA
GCTTTCCCTGCCCCCATCGAGAAAACCATCTCCAAAACCAAAGGCAGACCGAAGGCTCCACAGGTGTACACCATTCCACC
TCCCAAGGAGCAGATGGCCAAGGATAAAGTCAGTCTGACCTGCATGATAACAGACTTCTTCCCTGAAGACATTACTGTGG
AGTGGCAGTGGAATGGGCAGCCAGCGGAGAACTACAAGAACACTCAGCCCATCATGAACACGAATGGCTCTTACTTCGTC
TACAGCAAGCTCAATGTGCAGAAGAGCAACTGGGAGGCAGGAAATACTTTCACCTGCTCTGTGTTACATGAGGGCCTGCA
CAACCACCATACTGAGAAGAGCCTCTCCCACTCTCCTGGGCTGCAACTGGACGAGACCTGTGCTGAGGCCCAGGACGGGG
AGCTGGACGGGCTCTGGACGACCATCACCATCTTCATCAGCCTCTTCCTGCTCAGCGTGTGCTACAGCGCTGCTGTCACA
CTCTTCAAGGTAAAGTGGATCTTCTCCTCGGTGGTGGAGCTGAAGCAGACACTGGTTCCTGAATACAAGAACATGATTGG
GCAAGCGCCCTAGagatctggccggctgggcccgtttcgaaggtaagcctatccctaaccctctcctcggtctcgattct
acgcgtaccggtcatcatcaccatcaccattgagtttaaacccgctgatcagcctcgactgtgccttctagttgccagcc
atctgttgtttgcccctcccccgtgccttccttgaccctggaaggtgccactcccactgtcctttcctaataaaatgagg
aaattgcatcgcattgtctgagtaggtgtcattctattctggggggtggggtggggcaggacagcaagggggaggattgg
gaagacaatagcaggcatgctggggatgcggtgggctctatggcttctgaggcggaaagaaccagtggcggtaatacggt
tatccacagaatcaggggataacgcaggaaagaacatgtgagcaaaaggccagcaaaaggccaggaaccgtaaaaaggcc
gcgttgctggcgtttttccataggctccgcccccctgacgagcatcacaaaaatcgacgctcaagtcagaggtggcgaaa
cccgacaggactataaagataccaggcgtttccccctggaagctccctcgtgcgctctcctgttccgaccctgccgctta
ccggatacctgtccgcctttctcccttcgggaagcgtggcgctttctcatagctcacgctgtaggtatctcagttcggtg
taggtcgttcgctccaagctgggctgtgtgcacgaaccccccgttcagcccgaccgctgcgccttatccggtaactatcg
tcttgagtccaacccggtaagacacgacttatcgccactggcagcagccactggtaacaggattagcagagcgaggtatg
taggcggtgctacagagttcttgaagtggtggcctaactacggctacactagaaggacagtatttggtatctgcgctctg
ctgaagccagttaccttcggaaaaagagttggtagctcttgatccggcaaacaaaccaccgctggtagcggtggtttttt
tgtttgcaagcagcagattacgcgcagaaaaaaaggatctcaagaagatcctttgatcttttctacggggtctgacgctc
agtggaacgaaaactcacgttaagggattttggtcatgacattaacctataaaaataggcgtatcacgaggccctttcgt
ctcgcgcgtttcggtgatgacggtgaaaacctctgacacatgcagctcccggagacggtcacagcttgtctgtaagcgga
tgccgggagcagacaagcccgtcagggcgcgtcagcgggtgttggcgggtgtcggggctggcttaactatgcggcatcag
agcagattgtactgagagtgcaccatatatgcggtgtgaaataccgcacagatgcgtaaggagaaaataccgcatcaggc
gccattcgccattcaggctgcgcaactgttgggaagggcgatcggtgcgggcctcttcgctattacgcca
3'
```

pmIgαβ: Sequence that corresponds to map shown in figure 1B. In capital letters are depicted the Igα and the Igβ exons.

```
gcgcgcgttgacattgattattgactagttattaatagtaatcaattacggggtcattagttcatagcccatatatggag
ttccgcgttacataacttacggtaaatggcccgcctggctgaccgcccaacgacccccgcccattgacgtcaataatgac
gtatgttcccatagtaacgccaatagggactttccattgacgtcaatgggtggactatttacggtaaactgcccacttgg
cagtacatcaagtgtatcatatgccaagtacgccccctattgacgtcaatgacggtaaatggcccgcctggcattatgcc
cagtacatgaccttatgggactttcctacttggcagtacatctacgtattagtcatcgctattaccatggtgatgcggtt
ttggcagtacatcaatgggcgtggatagcggtttgactcacggggatttccaagtctccaccccattgacgtcaatggga
gtttgttttggcaccaaaatcaacgggactttccaaaatgtcgtaacaactccgccccattgacgcaaatgggcggtagg
cgtgtacggtgggaggtctatataagcagagctctctggctaactagagaacccactgcttactggcttatcgaaattaa
tacgactcactatagggagacccaagcttgccaccATGCCAGGGGGTCTAGAAGCCCTCAGAGCCCTGCCTCTCCTCCTC
TTCTTGTCATACGCCTGTTTGGGTCCCGGATGCCAGGCCCTGCGGGTAGAAGGGGGTCCACCATCCCTGACGGTGAACTT
GGGCGAGGAGGCCCGCCTCACCTGTGAAAACAATGGCAGGAACCCTAATATCACATGGTGGTTCAGCCTTCAGTCTAACA
TCACATGGCCCCCAGTGCCACTGGGTCCTGGCCAGGGTACCACAGGCCAGCTGTTCTTCCCCGAAGTAAACAAGAACCAC
AGGGGCTTGTACTGGTGCCAAGTGATAGAAAACAACATATTAAAACGCTCCTGTGGTACTTACCTCCGCGTGCGCAATCC
AGTCCCTAGGCCCTTCCTGGACATGGGGGAAGGTACCAAGAACCGCATCATCACAGCAGAAGGGATCATCTTGCTGTTCT
GTGCAGTGGTGCCAGGGACGCTGCTGCTATTCAGGAAACGGTGGCAAAATGAGAAGTTTGGGGTGGACATGCCAGATGAC
TATGAAGATGAAAATCTCTATGAGGGCCTGAACCTTGATGACTGTTCTATGTATGAGGACATCTCCAGGGGACTCCAGGG
CACCTACCAGGATGTGGGCAACCTCCACATTGGAGATGCCcagctggaaaagccatgaggatccgaacaaaaactcatct
cagaagaggatctgaatatgcataccggtcatcatcaccatcaccattgagtttgatccccgggaattCagacatgataa
gatacattgatgagtttggacaaaccacaactagaatgcagtgaaaaaaatgctttatttgtgaaatttgtgatgctatt
gctttatttgtaaccattataagctgcaataaacaagttggggtgggcgaagaactccagcatgagatccccgcgctgga
ggatcatccagccggcgtcccggaaaacgattccgaagcccaacctttcatagaaggcggcggtggaatcgaaatctcgt
agcacgtgtcagtcctgctcctcggccacgaagtgcacgcagttgccggccgggtcgcgcagggcgaactcccgcccca
cggctgctcgccgatctcggtcatggccggcccggaggcgtcccggaagttcgtggacacgacctccgaccactcggcgt
acagctcgtccaggccgcgcacccacacccaggccagggtgttgtccggcaccacctggtcctggaccgcgctgatgaac
agggtcacgtcgtcccggaccacaccggcgaagtcgtcctccacgaagtcccgggagaacccgagccggtcggtccagaa
ctcgaccgctccggcgacgtcgcgcgcggtgagcaccggaacggcactggtcaacttggccatggtttagttcctcacct
tgtcgtattatactatgccgatatactatgccgatgattaattgtcaacacgtgctgatcagatccgaaaatggatatac
aagctcccgggagcttttgcaaaagcctaggcctccaaaaaagcctcctcactacttctggaatagctcagaggcagag
gcggcctcggcctctgcataaataaaaaaaattagtcagccatggggcggagaatgggcggaactgggcggagttagggg
cgggatgggcggagttaggggcgggactatggttgctgactaattgagatgcatgctttgcatacttctgcctgctgggg
agcctggggactttccacacctggttgctgactaattgagatgcatgctttgcatacttctgcctgctggggagcctggg
gactttccacaccctcgtcgagctagcttcgtgaggctccggtgcccgtcagtgggcagagcgcacatcgcccacagtcc
ccgagaagttggggggaggggtcggcaattgaaccggtgcctagagaaggtggcgcggggtaaactgggaaagtgatgtc
gtgtactggctccgcctttttcccgagggtgggggagaaccgtatataagtgcagtagtcgccgtgaacgttctttttcg
caacgggtttgccgccagaacacaggtaagtgccgtgtgtggttcccgcgggcctggcctctttacgggttatggccctt
```

gcgtgccttgaattacttccacctggctccagtacgtgattcttgatcccgagctggagccaggggcgggccttgcgctt
taggagccccttcgcctcgtgcttgagttgaggcctggcctgggcgctggggccgccgcgtgcgaatctggtggcacctt
cgcgcctgtctcgctgctttcgataagtctctagccatttaaaatttttgatgacctgctgcgacgctttttttctggca
agatagtcttgtaaatgcgggccaggatctgcacactggtatttcggtttttgggcccgcggccggcgacggggcccgtg
cgtcccagcgcacatgttcggcgaggcggggcctgcgagcgcggccaccgagaatcggacgggggtagtctcaagctggc
cggcctgctctggtgcctggcctcgcgccgccgtgtatcgccccgccctgggcggcaaggctggcccggtcggcaccagt
tgcgtgagcggaaagatggccgcttcccggccctgctccagggggctcaaaatggaggacgcggcgctcgggagagcggg
cgggtgagtcacccacacaaaggaaaagggcctttccgtcctcagccgtcgcttcatgtgactccacggagtaccgggcg
ccgtccaggcacctcgattagttctggagcttttggagtacgtcgtctttaggttggggggaggggttttatgcgatgga
gtttccccacactgagtgggtggagactgaagttaggccagcttggcacttgatgtaattctcgttggaatttgcccttt
ttgagtttggatcttggttcattctcaagcctcagacagtggttcaaagtttttttcttccatttcaggtgtcgtgaaca
cgtggtcgcggccgcaccATGGCCACACTGGTGCTGTCTTCCATGCCCTGCCACTGGCTGTTGTTCCTGCTGCTGCTCTT
CTCAGGTGAGCCGGTACCAGCAATGACAAGCAGTGACCTGCCACTGAATTTCCAAGGAAGCCCTTGTTCCCAGATCTGGC
AGCACCCGAGGTTTGCAGCCAAAAAGCGGAGCTCCATGGTGAAGTTTCACTGCTACACAAACCACTCAGGTGCACTGACC
TGGTTCCGAAAGCGAGGGAGCCAGCAGCCCCAGGAACTGGTCTCAGAAGAGGGACGCATTGTGCAGACCCAGAATGGCTC
TGTCTACACCCTCACTATCCAAAACATCCAGTACGAGGATAATGGTATCTACTTCTGCAAGCAGAAATGTGACAGCGCCA
ACCATAATGTCACCGACAGCTGTGGCACGGAACTTCTAGTCTTAGGATTCAGCACGTTGGACCAACTGAAGCGGCGGAAC
ACACTGAAAGATGGCATTATCTTGATCCAGACCCTCCTCATCATCCTCTTCATCATTGTGCCCATCTTCCTGCTACTTGA
CAAGGATGACGGCAAGGCTGGGATGGAGGAAGATCACACCTATGAGGGCTTGAACATTGACCAGACAGCCACCTATGAAG
ACATAGTGACTCTTCGGACAGGGGAGGTAAAGTGGTCGGTAGGAGAGCATCCAGGCCAGGAATGActcgaggagatctgg
ccggctgggcccgtttcgaaggtaagcctatccctaaccctctcctcggtctcgattctacgcgtaccggtcatcatcac
catcaccattgagtttaaacccgctgatcagcctcgactgtgccttctagttgccagccatctgttgtttgcccctcccc
cgtgccttccttgaccctggaaggtgccactcccactgtcctttcctaataaaatgaggaaattgcatcgcattgtctga
gtaggtgtcattctattctggggggtggggtggggcaggacagcaagggggaggattgggaagacaatagcaggcatgct
ggggatgcggtgggctctatggcttctgaggcggaaagaaccagtggcggtaatacggttatccacagaatcaggggata
acgcaggaaagaacatgtgagcaaaaggccagcaaaaggccaggaaccgtaaaaaggccgcgttgctggcgtttttccat
aggctccgcccccctgacgagcatcacaaaaatcgacgctcaagtcagaggtggcgaaacccgacaggactataaagata
ccaggcgtttccccctggaagctccctcgtgcgctctcctgttccgaccctgccgcttaccggatacctgtccgcctttc
tcccttcgggaagcgtggcgctttctcatagctcacgctgtaggtatctcagttcggtgtaggtcgttcgctccaagctg
ggctgtgtgcacgaaccccccgttcagcccgaccgctgcgccttatccggtaactatcgtcttgagtccaacccggtaag
acacgacttatcgccactggcagcagccactggtaacaggattagcagagcgaggtatgtaggcggtgctacagagttct
tgaagtggtggcctaactacggctacactagaaggacagtatttggtatctgcgctctgctgaagccagttaccttcgga
aaaagagttggtagctcttgatccggcaaacaaaccaccgctggtagcggtggtttttttgtttgcaagcagcagattac
gcgcagaaaaaaaggatctcaagaagatcctttgatcttttctacggggtctgacgctcagtggaacgaaaactcacgtt
aagggattttggtcatgacattaacctataaaaataggcgtatcacgaggccctttcgtctcgcgcgtttcggtgatgac
ggtgaaaacctctgacacatgcagctcccggagacggtcacagcttgtctgtaagcggatgccgggagcagacaagcccg
tcagggcgcgtcagcgggtgttggcgggtgtcggggctggcttaactatgcggcatcagagcagattgtactgagagtgc
accatatatgcggtgtgaaataccgcacagatgcgtaaggagaaaataccgcatcaggcgccattcgccattcaggctgc
gcaactgttgggaagggcgatcggtgcgggcctcttcgctattacgcca
